# Transcranial Doppler to assess sepsis-associated encephalopathy in critically ill patients

**DOI:** 10.1186/1471-2253-14-45

**Published:** 2014-06-11

**Authors:** Charalampos Pierrakos, Rachid Attou, Laurence Decorte, Athanasios Kolyviras, Stefano Malinverni, Philippe Gottignies, Jacques Devriendt, David De Bels

**Affiliations:** 1 Departments of Intensive Care, Brugmann University Hospital, Université Libre de Bruxelles, Place Van Gehuchten 4, 1020 Bruxelles, Belgium; 2 Departments of Geriatrics, Brugmann University Hospital, Université Libre de Bruxelles, Bruxelles, Belgium

## Abstract

**Background:**

Transcranial Doppler can detect cerebral perfusion alteration in septic patients. We correlate static Transcranial Doppler findings with clinical signs of sepsis-associated encephalopathy.

**Methods:**

Forty septic patients were examined with Transcranial Doppler on the first and third day of sepsis diagnosis. The pulsatility index (PI) and cerebral blood flow index (CBFi) were calculated by blood velocity in the middle cerebral artery (cm/sec). Patients underwent a daily cognitive assessment with the Confusion Assessment Method for the Intensive Care Unit (CAM-ICU) test.

**Results:**

Twenty-one patients (55%) were found to present confusion. The majority of the patients presented a PI > 1.1 (76%). PI on the first day (but not the third day) could predict a positive CAM-ICU test in septic patients (PI cut-off: 1.3, AUC: 0.905, p < 0.01, sensitivity: 95%, specificity: 88%, AUC: 0.618, p = 0.24). Multivariable analysis showed that PI on the first day is related to a positive CAM-ICU test independent of age and APACHE II score (OR: 5.6, 95% CI: 1.1-29, p = 0.03). A decrease of the PI on the third day was observed in the group that presented initially high PI (>1.3) (2.2 ± 0.71 vs. 1.81 ± 0.64; p = 0.02). On the other hand, an increase in PI was observed in the other patients (1.01 ± 0.15 vs. 1.58 ± 0.57; p < 0.01). On only the first day, the mean blood velocity in the middle cerebral artery and CBFi were found to be lower in those patients with a high initial PI (36 ± 21 vs. 62 ± 28 cm/sec; p < 0.01, 328 ± 101 vs. 581 ± 108; p < 0.01, respectively).

**Conclusions:**

Cerebral perfusion disturbance observed with Transcranial Doppler could explain clinical symptoms of sepsis-associated encephalopathy.

## Background

Sepsis-associated encephalopathy (SAE) is a common complication in critically ill patients and is considered to be an independent prognostic factor for increased mortality [1,2]. The pathophysiology of SAE is still quite obscure [3,4].

Cerebral microcirculation alterations related to sepsis are characterized by a decrease in the density of perfused microvessels [5]. An increase in the distance between neurons and capillaries can possibly cause an inadequate oxygen supply. Given that the brain is highly dependent on an adequate oxygen supply, any deficit can be related to immediate important cerebral dysfunction [6]. Additionally, a decrease in the density of perfused microvessels can be related to an increase in cerebrovascular resistance. The pulsatility index (PI), as an indicator of cerebrovascular resistance, has been found to be higher in septic patients, compared with normal controls [7] or non-septic critically ill patients [8]. The aim of this study is to correlate the clinical presentation of SAE with changes in PI, as evaluated by Transcranial Doppler (TCD).

## Methods

This is a prospective, observational study conducted in our 33-bed intensive care unit (ICU) during a 4-month period (January-April 2013). Patients with sepsis for fewer than 24 hours were included in the study. Sepsis was defined according to standard international criteria [9]. Septic shock was defined as the need of noradrenaline support of more than 0.1 μg/kg/min. The Ethics Committee of the Brugmann University Hospital approved the study protocol and verbal consent was obtained from all patients (or from relatives when the patient was not conscious).

Exclusion criteria were: 1) an age less than 18 years old, 2) known cerebral lesions (ischemic or hemorrhagic cerebrovascular event, neoplasm), 3) cerebral infection, 4) patient support by Intra Aortic Balloon Pump or ECMO, 5) intoxication due to drugs, 6) known severe carotid stenosis (>70%), and 7) pregnancy.

We collected demographic information, such as length of stay in the ICU, source of sepsis, and relevant microbiological results. Clinical and laboratory data concerning organ failure were also compiled. The severity of illness was assessed with the Acute Physiology and Chronic Health Evaluation (APACHE) II score.

Blood velocity in the middle cerebral artery (VMCA) was measured with a 3-MHz TCD probe, going through the temporal bone window at both sides of the skull within the first day of sepsis for 10 seconds. The values of the brain side with the highest mean VMCA were registered. Three days later, the examination was repeated on the same side of the skull. At the time of the measurements, the patients were deemed to have a stable hemodynamic status. We calculated PI (PI = (velocity systolic-velocity diastolic)/mean velocity) [10] and cerebral blood flow index (CBFi = MAP x 10/1.47^PI^), where MAP stands for mean arterial pressure [8].

SAE is an acute-onset encephalopathy attributed to systematic inflammatory response to sepsis, characterized by a diffuse or multifocal brain dysfunction and a variety of clinical signs (e.g., seizures, coma, altered mental status) [11,12]. We chose to evaluate patients with the Confusion Assessment Method for the Intensive Care Unit (CAM-ICU) test as this method can evaluate the acute onset of neurological dysfunction and inattention, altered level of consciousness, and disorganized thinking, which can be symptoms related to SAE [13]. In order to assess early cerebral microcirculation alterations as a cause of cerebral dysfunction, we tested the predictive value of PI on the first or third day for a positive CAM-ICU test. Given that we evaluated static PI, we expected that an increased PI will be related to relative prolonged microcirculation disturbances. Therefore, we expected that neurological symptoms might be persistent or even presented later than the initial measurement. For this reason, patients were evaluated via the CAM-ICU test, once per day (between 11:00–14:00), during their stay in the ICU, or for 10 days. Sedated patients were evaluated at least 6 hours after the cessation of medication.

### Statistical analysis

Statistical analysis was performed with SPSS software (SPSS Inc., Chicago, IL, USA). Receiver operating characteristic (ROC) curve analysis was used to assess the effectiveness of PI on the first and third day – in order to predict delirium and determine a cut-off value with optimal sensitivity and specificity. According to the cut-off value, first-day patients were divided into two groups: Group A (PI higher than the cut-off value) and Group B (PI lower than the cut-off value). A Kolomogorov-Smirnov test was used to verify the normality of the distribution of continuous variables. The Student’s t-test was used for continuous variables, while categorical variables were compared with Fisher’s exact test. Pearson’s correlation was applied to evaluate the relationships between PI and age. Given that age and severity of the patients are two risk factors for delirium that can possibly affect PI too, we performed multivariable logistic regression analysis to evaluate PI as an independent risk factor for a positive CAM-ICU test. For this model the minimum required sample size, for *f*^2^ = 0.35 (anticipated effect size) and desired statistical power level 0.8, was found to be 36 patients. Statistical significance was defined as p < 0.05.

## Results

Forty patients with sepsis were included in the study, while two patients were excluded, since the physicians questioned the diagnosis of sepsis in those cases. Pulmonary infection was the source of infection in the majority of patients (64%). Gram-negative pathogens were responsible for sepsis in 46% of septic patients. The majority of patients (76%) presented a maximum PI > 1.1.

Twenty-one patients (55%) presented delirium (positive CAM-ICU test). Sixteen patients presented a positive CAM-ICU test on the first study day. Three patients could not be evaluated on the first day because of sedation. Two of these patients were initially evaluated three days later for the first time and one was evaluated five days later, when a positive CAM-ICU test was found. Two patients showed a negative CAM-ICU test on the first day and a positive test on the second study day.

**Figure 1:**
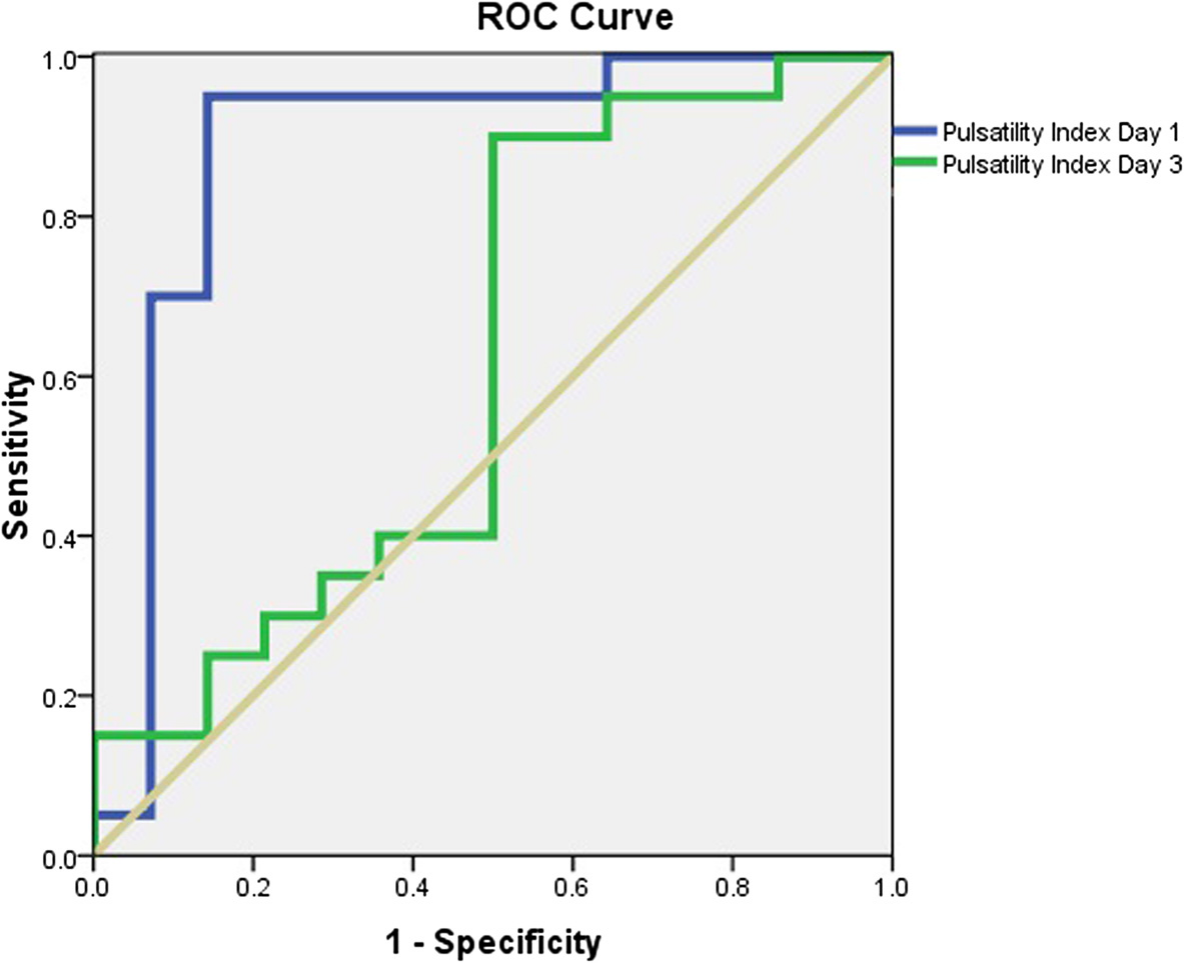
**ROC curves for PI on the first day (blue line) and third day (green line).** AUC were 0.908 and 0.618, respectively.

ROC curve analysis showing only PI on the first day and not the third day was a good predictor of the presence of confusion (AUC = 0.908, 95%, CI 0.80-0.98, p < 0.01 and AUC = 0.618, 95% CI 0.44-0.791, p = 0.24, respectively; Figure 1). For a cut-off value of 1.3, we found a 95% sensitivity and an 88% specificity. We found no significant correlation between PI and age on the first or third days (r^2^ = 0.09, p = 0.06; r^2^ = 0.05, p = 0.16, respectively). Multivariate logistic regression analysis showed that PI was related to confusion, independent of age and APACHE II score (OR: 5.66, 95% CI: 1.1-29.11, p = 0.03; Table 1).

**Table 1:** Multivariable logistic regression analysis with positive CAM-ICU test as the dependent variable

	**OR (95% CI)**	**p value**
**Age**	1.02 (0.95-1.09)	0.56
**APACHE II score**	1.19 (0.95-1.49)	0.12
**PI**	5.66 (1.12-29.11)	0.03

PI: pulsatility index on the first day.

Demographic characteristics of the patients are presented in Table 2. Patients with high PI (>1.3) were older (72 ± 13 vs. 62 ± 16 years, p = 0.04) with higher APACHE II scores (23 ± 5 vs. 18 ± 5, p < 0.01). The prevalence of septic shock was greater in patients with high PI (63% vs. 25%, p = 0.01).

**Table 2:** Patients’ demographic characteristics

	**PI < 1.29**	**PI > 1.3**	**p values**
**No. of patients**	16	22	
**Age (Years)**	62 ± 16	72 ± 13	0.04
**Type of admission**			
Medical (%)	11 (69)	16 (73)	0.78
**APACHE II Score**	18 ± 5	23 ± 5	<0.01
**Septic shock (%)**	4 (25)	14 (63)	0.01
**Mechanical Ventilation (%)**	6 (37)	14 (64)	0.11
**GCS**^**1**^	14 ± 1	12 ± 3	0.02
**Delirium**^**2**^**(%)**	2 (12)	19 (86)	<0.01
**CRP**^3^**(mg/dl)**	24 ± 15	20 ± 10	0.41
**Hg (g/dl)**	9.4 ± 2	10 ± 2	0.34
**ICU LOS**^**4**^**(days)**	5 (3–10)	11 (3–60)	0.04
**ICU mortality (%)**	3 (18)	8 (36)	0.23
**Hospital mortality (%)**	3 (18)	10 (45)	0.08

^1^Glasgow Coma Score at the time the sepsis diagnosis was made.

^2^Patients with a positive CAM-ICU test at any time during the study period.

^3^C-reactive protein, maximum value within the first 48 h.

^4^Intensive care unit length of stay.

**Figure 2:**
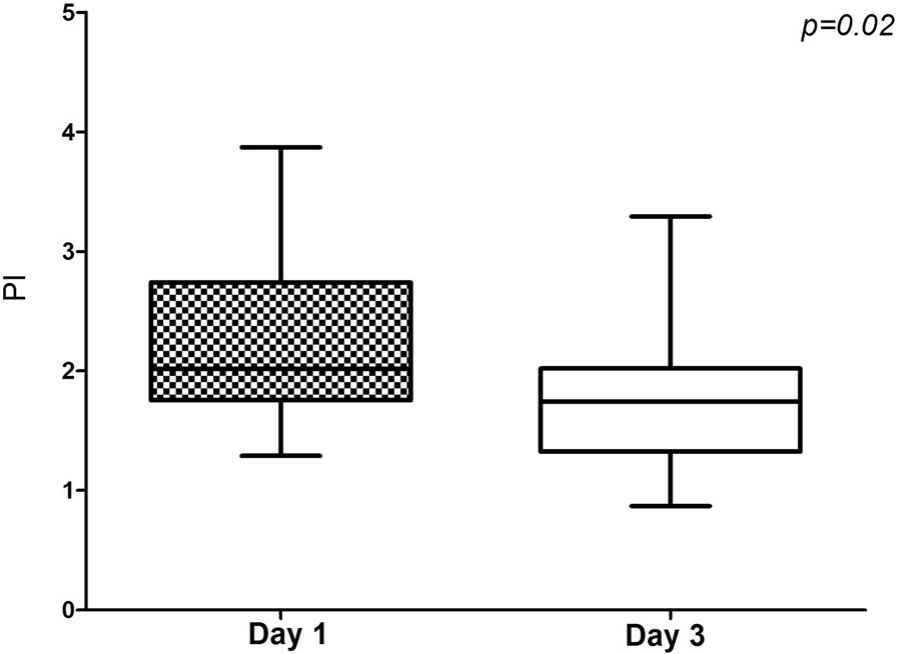
PI evolution in patients who showed PI > 1.3 on the first day.

**Figure 3:**
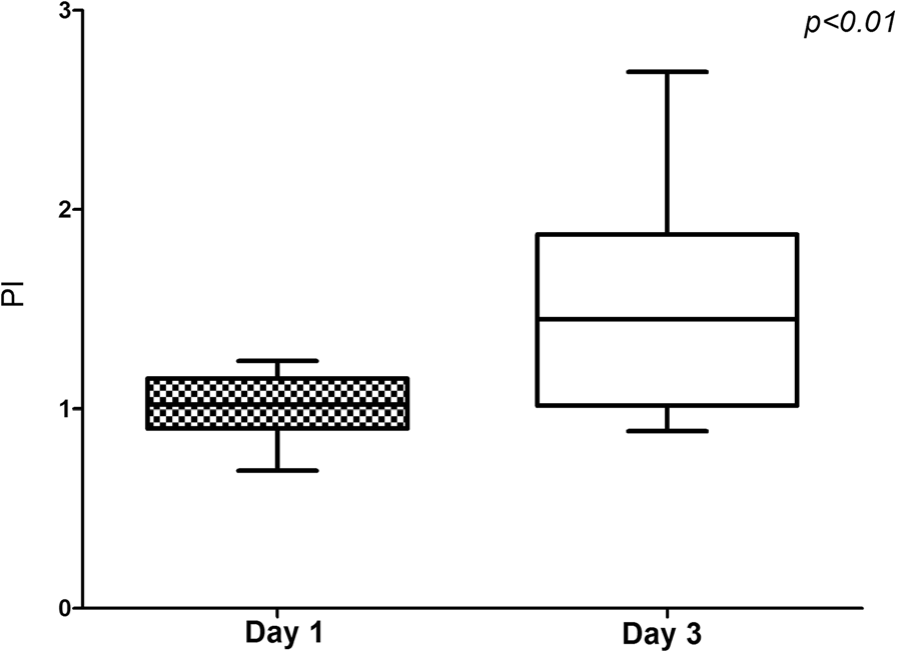
PI evolution in patients who showed PI < 1.3 on the first day.

The measurements of VMCA and calculations of PI and CBFi, as well as MAP and pCO_2_ values at the time of examination, are presented in Tables 3 and 4. Patients with high PI have statistically lower values of mean VMCA and CBFi compared with patients with lower PI (36 ± 21 vs. 62 ± 28 cm/sec, p < 0.01; 328 ± 101 vs. 581 ± 108, p < 0.01), only on the first day. No statistically significant differences for PI were found between groups on the third day (1.81 ± 0.64 vs. 1.58 ± 0.57). The evolution of PI over three days of observation is presented in Figures 2 and 3.

**Table 3:** Day 1 data of TCD measurements

	**PI < 1.29**	**PI > 1.3**	**p values**
VMCA systolic (cm/sec)	103 ± 47	92 ± 44	0.48
VMCA diastolic (cm/sec)	40 ± 20	16 ± 13	< 0.01
VMCA mean (cm/sec)	62 ± 28	36 ± 21	< 0.01
PI	1.01 ± 0.15	2.2 ± 0.71	< 0.01
CBFi	581 ± 108	328 ± 101	< 0.01
pCO_2_ (mmHg)	40 ± 13	37 ± 8	0.41
MAP (mmHg)	86 ± 12	75 ± 11	0.01

VMCA: velocity in middle cerebral artery, PI: pulsatility index, CBFi: cerebral blood flow index, MAP: mean arterial pressure.

**Table 4:** Day 3 data of TCD measurements

	**PI < 1.29**	**PI > 1.3**	**p values**
VMCA systolic (cm/sec)	85 ± 47	95 ± 46	0.56
VMCA diastolic (cm/sec)	25 ± 21	22 ± 17	0.61
VMCA mean (cm/sec)	45 ± 30	44 ± 24	0.91
PI	1.58 ± 0.57	1.81 ± 0.64	0.32
CBFi	440 ± 131	424 ± 115	0.71
pCO_2_ (mmHg)	43 ± 9	43 ± 7	0.91
MAP (mmHg)	78 ± 12	83 ± 10	0.31

VMCA: velocity in middle cerebral artery, PI: pulsatility index, CBFi: cerebral blood flow index, MAP: mean arterial pressure.

## Discussion

The most important finding of this study is that PI measured within the first 24 hours after sepsis initiation is related to clinical signs of SAE. The value of PI of 1.3 represents a cut-off point that can be used in clinical practice. However, PI measured more than 72 hours after sepsis initiation is not related to the presence of delirium in septic patients.

PI is a parameter that is commonly used to describe Doppler waves and it is positively related to changes in vascular peripheral resistance [14]. The relationship of cerebral PI and peripheral cerebral resistances was doubted, as in cases of acute decrease in cerebral perfusion pressure because of the increase in intracranial pressure, an increase of PI is observed despite compensating cerebral vasodilation [15-17]. We evaluate static PI and not acute changes of PI; patients were hemodynamically stable at the time of examination with expected cerebral perfusion pressure much higher than the lower limit of autoregulation. Experimental [16] and clinical studies [17,18] showed that PI is a good reflection of changes in peripheral cerebrovascular resistances when cerebral perfusion pressure is stable. Increased static PI is found to be associated with angiographically demonstrated diffuse intracranial vessel disease in hemodynamically stable patients [19]. Consequently, we believe that the increase of PI that we found in patients with delirium corresponds to alterations of cerebral microcirculation.

Cerebral microcirculation is supposed to play an important role in the initiation of SAE, but also represents an anatomical structure that is possibly being attacked during sepsis. Cerebral endothelial cells are activated early in sepsis and they release pro-inflammatory cytokines and nitric monoxide in the brain, initiating or aggravating cerebral inflammation [20,21]. On the other hand, edema associated with cerebral inflammation [22], neutrophil [23], and platelet accumulation [24] within the cerebral microvessels impairs microcirculation. In our study, the majority of patients also showed a high PI at some point during their ICU stay. Given that increased PI is associated with increased cerebral resistance, we suspect that the majority of patients showed cerebral microcirculation impairment. Interestingly, the time of the microcirculation change was not homogeneous, with some patients showing changes early in the course of sepsis and others showing changes later (after the third day; Figures 2 and 3).

Even though previous studies have shown disturbances of cerebral microcirculation [25], its relation to cerebral function has not yet been well evaluated. A decrease of oxygen transport because of microcirculation disturbances could possibly counterbalanced by a decrease of cerebral metabolic needs [26]. Experimental studies have shown that cerebral microcirculation disturbances precede neuronal dysfunction in sepsis [27]. Cerebrovascular autoregulation disturbances were found only in septic patients with delirium [28]. Visually evoked potentials in patients with community-acquired pneumonia, evaluated in the acute phase, were similar to normal controls when autoregulatory disturbances were present [29]. The results of our study show that the relation between clinical signs of SAE and cerebral microcirculation is more complicated since we found that only high PI (>1.3) early in the course of sepsis is related to clinical symptoms of SAE. The group of patients that exhibited high PI on the first day also showed a lower Glasgow coma score (GCS) at the initiation of sepsis. Independently of the reason (e.g., hypotension, inflammation, etc.), this fact may imply a more severe affection of cerebral tissue that can cause significant early cerebral microcirculatory disturbances. It is possible that impairment of oxygen transport only in the early phases of sepsis is related to functional cerebral alterations.

Of the microcirculation disturbances, decrease of global cerebral blood may also contribute to the pathophysiology of SAE [4]. Increased cerebrovascular resistance and autoregulatory disturbances may expose septic patients to a decrease in cerebral blood flow (CBF) if the decrease is not compensated for by an increase in cerebral perfusion pressure. An experimental study showed that after 18 hours from the onset of sepsis, only animals with MAP values less than 65 mmHg showed cerebral hypoxia (lactate/pyruvate > 40), even though they showed similar functional cerebral capillary density and proportions of small perfused cerebral vessels compared with animals with higher MAP values (65-70 mmHg) [30]. In our study, patients with high PI showed a lower mean VMCA and CBFi compared with patients with lower PI. The lower MAP and coexistence increased resistance, potentially resulting in a decrease in CBF in these patients. Our results support the premise that a decrease in CBF plays an important role in the presence of delirium. Conversely, a potential increase of perfusion pressure may increase CBF and may therefore represent a therapeutic target in these patients.

The present study has some limitations. First, the study was not blinded: the TCD assessor was aware of the clinical image of the patient, as well as the results of the CAM-ICU test. However, PI does not depend on the insonation angle applied by the assessor [31] and patient assessment with the CAM-ICU test was performed by an occupational therapist who was unaware of the TCD results. In this manner, any bias was minimized. Patients with PI > 1.3 were not totally analogous to patients with lower PI as they showed a higher age and higher APACHE II score compared with patients with lower PI. As a result, we cannot exclude the possibility that high PI is a co-founder. However, we think that these two factors cannot explain our results since a multivariable analysis that evaluate age, APACHE II score, and PI as risk factors for a positive CAM-ICU test revealed PI as an independent risk factor. Additionally, we did not find any correlation between PI and age. As we tried to evaluate the PI values in an easily applicable clinical relevant context, we measured VMCA for only limited time (10 sec). However given that we evaluated patients in stable hemodynamic and respiratory conditions we think that this has only minor effect on our results. We only assessed TCD findings in relation to clinical symptoms associated to SAE, but not with any neuroradiological or neurophysiological results (e.g., electroencephalograms or magnetic resonance imaging). Consequently, we cannot confirm the normality of patients with PI < 1.3.

## Conclusions

Our results show that cerebral vascular constriction, detected by TCD in the early stages of sepsis, is correlated with clinical signs of SAE. A cut-off value of PI > 1.3 could be used in clinical practice as a risk factor for delirium in septic patients. The evolution of cerebral perfusion could vary between patients during the course of sepsis, which might then be followed up by TCD. Further studies are warranted to confirm the results of our studies and evaluate the utility of TCD to guide therapies applied in septic patients.

### Key points

● Increased cerebral PI, measured within 24 hours after the start of antibiotics, is related to clinical signs of SAE.

● TCD is an efficient method to evaluate cerebral perfusion in critically ill, septic patients.

## Competing interests

On behalf of all authors, the corresponding author states that there is no conflict of interests.

## Authors’ contributions

CP: Conceived of the study, participated in study design, acquisition of data, performed statistical analysis, drafted the manuscript; RA, AK, SM: Participated in acquisition of data; LD: Performed CAM-ICU tests; PG, JD: Participated in study design; DDB: Coordinated the study , participated in study design, supervised the manuscript draft. All authors read and approved the final manuscript.

## Pre-publication history

The pre-publication history for this paper can be accessed here:

http://www.biomedcentral.com/1471-2253/14/45/prepub
